# Pharmacokinetics of Sativex^®^ in Dogs: Towards a Potential Cannabinoid-Based Therapy for Canine Disorders

**DOI:** 10.3390/biom10020279

**Published:** 2020-02-11

**Authors:** María Fernández-Trapero, Carmen Pérez-Díaz, Francisco Espejo-Porras, Eva de Lago, Javier Fernández-Ruiz

**Affiliations:** 1 Departamento de Medicina y Cirugía Animal, Facultad de Veterinaria, Universidad Complutense, 28040 Madrid, Spain; mftrap@gmail.com (M.F.-T.); cperezdiaz@vet.ucm.es (C.P.-D.); 2 Instituto Universitario de Investigación en Neuroquímica, Departamento de Bioquímica y Biología Molecular, Facultad de Medicina, Universidad Complutense, 28040 Madrid, Spain; fespejo@med.ucm.es (F.E.-P.); elagofem@med.ucm.es (E.d.L.); 3 Centro de Investigación Biomédica en Red de Enfermedades Neurodegenerativas (CIBERNED), 28031 Madrid, Spain; 4 Instituto Ramón y Cajal de Investigación Sanitaria (IRYCIS), 28034 Madrid, Spain

**Keywords:** pharmacokinetics, sublingual delivery, Sativex^®^, cannabidiol, Δ^9^-tetrahydrocannabinol, naïve dogs

## Abstract

The phytocannabinoid-based medicine Sativex^®^ is currently marketed for the treatment of spasticity and pain in multiple sclerosis patients and is being investigated for other central and peripheral pathological conditions. It may also serve in Veterinary Medicine for the treatment of domestic animals, in particular for dogs affected by different pathologies, including human-like pathological conditions. With the purpose of assessing different dosing paradigms for using Sativex in Veterinary Medicine, we investigated its pharmacokinetics when administered to naïve dogs via sublingual delivery. In the single dose arm of the study, adult Beagle dogs were treated with 3 consecutive sprays of Sativex, and blood samples were collected at 12 intervals up to 24 h later. In the multiple dose arm of the study, Beagle dogs received 3 sprays daily for 14 days, and blood samples were collected for 24 h post final dose. Blood was used to obtain plasma samples and to determine the levels of cannabidiol (CBD), Δ^9^-tetrahydrocannabinol (Δ^9^-THC) and its metabolite 11-hydroxy-Δ^9^-THC. Maximal plasma concentrations of both Δ^9^-THC (C_max_ = 18.5 ng/mL) and CBD (C_max_ = 10.5 ng/mL) were achieved 2 h after administration in the single dose condition and at 1 h in the multiple dose treatment (Δ^9^-THC: C_max_ = 24.5 ng/mL; CBD: C_max_ = 15.2 ng/mL). 11-hydroxy-Δ^9^-THC, which is mainly formed in the liver from Δ^9^-THC, was almost undetected, which is consistent with the use of sublingual delivery. A potential progressive accumulation of both CBD and Δ^9^-THC was detected following repeated exposure, with maximum plasma concentrations for both cannabinoids being achieved following multiple dose. Neurological status, body temperature, respiratory rate and some hemodynamic parameters were also recorded in both conditions, but in general, no changes were observed. In conclusion, this study demonstrates that single or multiple dose sublingual administration of Sativex to naïve dogs results in the expected pharmacokinetic profile, with maximal levels of phytocannabinoids detected at 1–2 h and suggested progressive accumulation after the multiple dose treatment.

## 1. Introduction

The endocannabinoid system, formed by endogenous ligands (anandamide, 2-arachidonoylglycerol), their receptors (cannabinoid receptor type-1 (CB_1_), cannabinoid receptor type-2 (CB_2_), possibly G-protein receptor 55 (GPR55) and others) and their enzymes for synthesis and degradation, has been largely associated to the pathogenesis and potential therapies of numerous central and peripheral pathologies [1,2,3]. The discovery, and biochemical and physiological characterization of this modulatory system was initiated at the end of the 1980s [4,5] and continued up to the next two decades [6,7,8], but its pharmacological relevance originated long before this, with the discovery of endocannabinoids and their receptors giving support to the old and current uses of cannabis plant and their derived compounds for therapy [9,10,11]. A key step in this process was the formulation and approval of the phytocannabinoid-based medicine Sativex^®^ (GW Pharmaceuticals, Cambridge, UK) for the first time in 2005 [12]. It was marketed for the treatment of spasticity and pain in multiple sclerosis patients [12,13,14], and it is being investigated for other central and peripheral pathological conditions in humans [15,16,17,18]. It may also serve in Veterinary Medicine for the treatment of domestic animals, in particular for dogs affected by different pathological conditions. This may include pathologies as osteoarthritis [19], atopic dermatitis [20], epilepsy [21], degenerative myelopathy [22], some neuroinflammatory diseases (meningitis-asteritis and intraspinal spirocercosis) [23], and others [24]. It is important to remark that the information collected in studies with dogs with these pathologies is not only important for the development of Sativex in Veterinary Medicine but also for their equivalents in the human pathologies, thus representing suitable translational models for studying specific human pathologies. For example, degenerative myelopathy is a multisystem central and peripheral axonopathy described in dogs in 1973 [25], with an overall prevalence of 0.19% [26], that shares pathogenic mechanisms with some forms of human amyotrophic lateral sclerosis, including mutations in superoxide dismutase type-1 (SOD-1, one of the major causes of the human disease) [27]. It is also important to remark that most elements of the endocannabinoid system have been found in canine tissues and cells with functions, in general, equivalent to humans. For example, the CB_1_ receptors are highly expressed in both central and peripheral nervous system in dogs playing similar neuromodulatory functions and with a spatial distribution that mostly overlaps the one found in humans [28]. This cross-species similarity is also apparent for CB_2_ receptor and fatty acid amide hydrolase (FAAH), and other indirect endocannabinoid-related components, including GPR55, peroxisome proliferator-activated receptors (PPAR) and transient receptor potential vanilloid type-1 (TRPV_1_) [29,30]. This is an important observation in view of a possible development of Sativex for the treatment of several canine pathologies. Such development should be initiated by conducting pharmacokinetic studies, which are currently lacking in dogs. There is only a recent study carried out in dogs with different doses of 1.08:1 ratio of Δ^9^-THC and CBD extracts, but they were administered orally [31] not via sublingual delivery. In addition, the study was not specifically aimed at investigating in depth the pharmacokinetic properties of a Sativex treatment in dogs but its possible adverse effects by analyzing its proconvulsant activity in these dogs in comparison to other animal species [31]. Therefore, with the purpose of determining the best dosage and timing for using Sativex in dogs affected by different pathologies, we designed the present study to investigate the pharmacokinetics of Sativex when administered using single or multiple administrations to naïve, healthy dogs via sublingual delivery, which is the classic route used for this medicine in humans.

## 2. Materials and Methods 

### 2.1. Animals and Treatments

Beagle dogs (3 males and 3 females) were housed in the animal facilities of the Faculty of Veterinary, Complutense University (Madrid, Spain) and fed with a standard diet (Science Plan™ Canine Adult Advanced Fitness™ Medium with Chicken^®^; Hill’s Pet Nutrition, Madrid, Spain). They had a weight in the range 11–13 kg and an age of 8.0 ± 0.8 months at the time of study. Animals were routinely subjected to clinical analysis to confirm adequate health status for use in experiments. They were housed individually from 24 h prior to the experiment and up to 36 h after and were food- and liquid-deprived for 12 and 2 h, respectively, prior to the onset of the treatment. Access to both food and liquid was reinstated 2 h post-administration. All experiments were conducted according to local and European rules (directive 2010/63/EU) and approved by the Committee for Animal Experimentation of our University.

In the single dose experiment, dogs were treated with 3 consecutive sprays of Sativex (equivalent to 8.1 mg of Δ^9^-THC and 7.5 mg of CBD), provided by GW Research Ltd. (Cambridge, UK) and blood was collected from the jugular vein under anesthesia in Aquisel® LH/Li HEPARIN tubes (Aquisel, Barcelona, Spain) at different times (0, 14, 30, 45, 60, 120, 180, 240, 300, 360, 720 and 1440 min). In the multiple dose experiment, dogs received 3 consecutive sprays (equivalent to 8.1 mg of Δ^9^-THC and 7.5 mg of CBD) daily at the same time over a 14-day period. Blood was collected every day just before the treatment (pre-dose) and 45 min (based on previous literature [11,12]) after treatment (post-dose), except on the last day where the same collection times were used as for the single dose treatment (0, 14, 30, 45, 60, 120, 180, 240, 300, 360, 720 and 1440 min). All tubes were centrifuged for 10 min at 2300 *g* immediately after collection, and the plasma was extracted, frozen on dry ice and stored at −70 °C until analysis.

### 2.2. Analysis of CBD, Δ^9^-THC and 11-Hydroxy-ΔΔ^9^-THC

The levels of CBD, Δ^9^-THC and its metabolite 11-hydroxy-Δ^9^-THC were quantified in the plasma samples. This analysis was carried out using a standardized procedure with LC-MS/MS equipment at Quotient Bioanalytical Sciences (LGC256463QB01 protocol; LGC Group, Cambridgeshire, UK) [32].

### 2.3. Recording of the Neurological Status, Body Temperature, Respiratory Rate and Hemodynamic Parameters

To determine the safety of the treatment with Sativex®, dogs were examined for possible neurological alterations using the Glasgow coma scale modified (GCSM) [33], as well as for possible changes in body temperature, respiratory rate and some hemodynamic parameters (systolic blood pressure and heart rate), using standard procedures specific for dogs that are currently available in the “Hospital Clínico Veterinario”, Complutense University (Madrid, Spain). These analyses were conducted on dogs from both protocols at 0, 60, 240, 360 and 720 min after the unique (single dose protocol) or the last (multiple dose protocol) administration.

### 2.4. Statistics

Data were normally distributed (tested with the Shapiro–Wilk normality test) and, in those presented in Figure 2, assessed by two-way ANOVA followed by the Bonferroni test to detect specific differences between groups, using GraphPad Prism^®^ software (version 5.01; GraphPad Software Inc., San Diego, CA, USA). The same software was used to determine the AUC for the three phytocannabinoids in the two dosing conditions (single and multiple). Data are presented as mean ± SEM.

## 3. Results

### 3.1. Levels of Cannabinoids after Sativex Treatment

**Figure 1:**
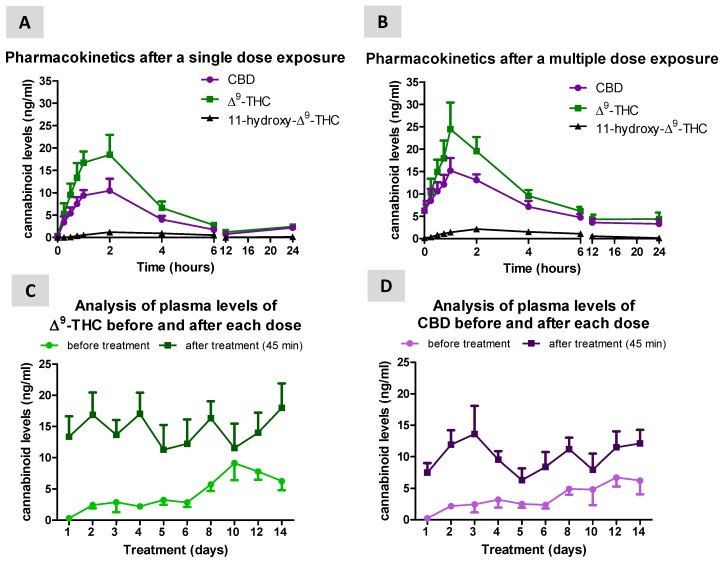
Plasma levels of CBD, Δ^9^-THC and its metabolite 11-hydroxy-Δ^9^-THC measured in naïve dogs at different times after a single dose (**A**) or multiple dose (**B**; 14 days) treatment with Sativex delivered by sublingual administration (3 consecutive sprays equivalent to 8.1 mg of Δ^9^-THC and 7.5 mg of CBD). Panels (**C**) and (**D**) represent the comparison of Δ^9^-THC and CBD levels before (pre-dose values) and after treatment (45 min; post-dose values) in the single and multiple dose conditions. Values are means ± SEM of 6 animals per group.

Following a single dose of 3 consecutive Sativex sprays, maximal levels of both Δ^9^-THC (C_max_ = 18.5 ng/mL) and CBD (C_max_ = 10.5 ng/mL) were reached at 2 h (t_max_) after administration (Figure 1A). AUC values in the single dose condition were 94.9 ng/mL × h for Δ^9^-THC and 60.4 ng/mL × h for CBD. A similar profile was seen following 14 days of daily treatment (multiple dose condition), but maximal plasma levels of both Δ^9^-THC (C_max_ = 24.5 ng/mL) and CBD (C_max_ = 15.2 ng/mL) were seen 1 h (t_max_) after administration (Figure 1B). AUC values in the multiple dose condition were higher compared to the single dose treatment, reaching 165.0 ng/mL × h for Δ^9^-THC and 123.1 ng/mL × h for CBD. 11-Hydroxy-Δ^9^-THC, which is mainly produced by the liver from Δ^9^-THC, was almost undetectable in concordance with the use of a sublingual delivery (Figure 1A,B), with values for AUC = 6.8 ng/mL × h, C_max_ = 1.2 ng/mL and t_max_ = 2 h in the single dose condition, and AUC = 18.2 ng/mL × h, C_max_ = 2.2 ng/mL and t_max_ = 2 h in the multiple dose treatment. A progressive numerical elevation of both Δ^9^-THC (Figure 1C) and CBD (Figure 1D) levels was also observed as the multiple dose treatment progressed, with the pre-dose and post-dose values tending to become progressively closer. This was, in general, due to the elevation of daily pre-dose values, in particular in the period between days 8^th^ until 14^th^. This was more evident for CBD (Figure 1D) than Δ^9^-THC (Figure 1C). 

### 3.2. Neurological, Temperature, Respiratory and Hemodynamic Parameters

**Figure 2:**
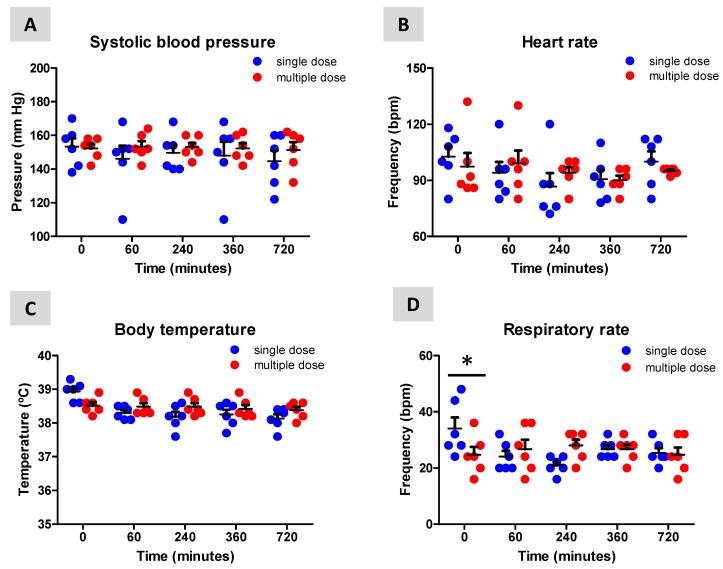
Effects of the single dose and multiple dose treatment with Sativex, delivered by sublingual administration (3 consecutive sprays equivalent to 8.1 mg of Δ^9^-THC and 7.5 mg of CBD), to naïve dogs on their systolic blood pressure (**A**), heart rate (**B**), body temperature (**C**) and respiratory rate (**D**). Values are means ± SEM of 6 animals per group. Data were assessed by using two-way ANOVA followed by the Bonferroni test (* *p* < 0.05 versus the single dose treatment).

Dogs were evaluated for possible neurological, temperature, respiratory and hemodynamic alterations during the two treatment conditions to determine whether treatment with Sativex was well-tolerated. No changes were detected in their neurological status (GCSM = 18 in all cases), systolic blood pressure (treatment: F(1,50) = 1.58, ns; interaction: F(4,50) = 0.202, ns; Figure 2A) and heart rate (treatment: F(1,50) = 0.01, ns; interaction: F(4,50) = 0.579, ns; Figure 2B) following single or multiple dose treatment with Sativex. However, body temperature (treatment: F(1,50) = 1.774, ns; interaction: F(4,50) = 3.234, *p* < 0.05; Figure 2C) and, in particular, respiratory rate (treatment: F(1,50) = 0.001, ns; interaction: F(4,50) = 3.023, *p* < 0.05; Figure 2D) were elevated at the beginning of the single dose treatment, which probably reflects the onset of manipulation of the dogs.

## 4. Discussion

The phytocannabinoid-derived medicine Sativex was licensed for the first time in 2005 in Canada for the treatment of spasticity and other neurological symptoms in multiple sclerosis patients and has been subsequently approved in over 25 countries for the treatment of spasticity due to multiple sclerosis [12], but it has also been proposed as a promising therapy for other pathological conditions in humans [9,10,11,15,16,17,18]. The use of Sativex could be also extended to Veterinary Medicine for the treatment of dogs and other domestic species affected by human-like or specific central and peripheral pathologies. Pharmacokinetic and pharmacodynamic properties of Sativex have been extensively investigated in humans and laboratory species (e.g., rodents) [34,35,36], but not in other species, such as dogs. The only study conducted with the 1.08:1 ratio of Δ^9^-THC and CBD extracts in dogs has been recently published [31], but it used an oral administration instead sublingual delivery, and, as has been mentioned above, rather than being a pharmacokinetic study, was aimed more at investigating the proconvulsant activity of different doses of the 1.08:1 ratio of Δ^9^-THC and CBD extracts in dogs in comparison to other animal species [31]. Given the interest to evaluate whether this phytocannabinoid-based medicine has beneficial effects on different central and peripheral canine disorders, the pharmacokinetic profile of Sativex, when administered via sublingual delivery to naïve dogs, has been determined. This may help to determine the best dosage and timing for the future evaluation of Sativex in dogs affected by different pathologies (e.g., osteoarthritis [19], atopic dermatitis [20], epilepsy [21], degenerative myelopathy [22], some neuroinflammatory diseases (meningitis-asteritis and intraspinal spirocercosis) [23], and others [24]), although the effect that different pathologies may have on Sativex pharmacokinetics remains unknown.

Our data indicated that Sativex was well-tolerated and did not produce any relevant effect on the neurological status, despite some neurological signs (e.g., ataxia) observed in the aforementioned study using different doses of the 1.08:1 ratio of Δ^9^-THC and CBD extracts administered orally to dogs [31]. However, it is important to remark that these signs were found at conditions that were significantly different (higher doses of the 1.08:1 ratio of Δ^9^-THC and CBD extracts and/or after more than 50 weeks of daily administration [31]) compared to those used in our present study, leading to plasma levels of approximately 200 ng/mL of CBD (20 fold higher than in our study) and 60 ng/mL of Δ^9^-THC (3 fold higher). In our study, no relevant effects were found for body temperature, respiratory function and hemodynamic values of the dogs, which, in general, are concordant (e.g., absence of tachypnoea) with the study conducted with different doses of the 1.08:1 ratio of Δ^9^-THC and CBD extracts administered orally to dogs [31]. Our data also indicated that the maximal levels in the plasma of the two abundant phytocannabinoids mixed in Sativex were achieved 2 h after the single dose treatment and earlier (at 1 h) after 14 days of daily administration. Our data also suggested a progressive elevation of both CBD and Δ^9^-THC levels as the multiple dose treatment progresses, with the pre-dose and post-dose values tending to become progressively closer. In general, this is the consequence of the elevation of daily pre-dose values. This potentially reflects their distribution and accumulation in fat tissues (and also in other organs) immediately after treatment and their progressive and slow release from these tissues when plasma levels tended to be significantly reduced (approximately 4 h after treatment) [37], although other explanations (e.g., reduced metabolism) cannot be ruled out. This fact, as well as the data obtained for C_max_ and t_max_ in our study are, in general, equivalent to the results obtained in pharmacokinetic studies conducted previously in laboratory rodents and, in particular, in humans [33,34,35]. Finally, our results also indicated that the major Δ^9^-THC metabolite, 11-hydroxy-Δ^9^-THC, which is mainly formed in the liver by the action of the cytochrome P450 complex, presented at very low levels in the plasma, and this should be also the case with some classic metabolites of CBD (e.g., 7-hydroxy-CBD), despite they were not analyzed in this study. This is in agreement with the use of a sublingual delivery for administration; furthermore, the well-known food effect on phytocannabinoid absorption may also be a contributing factor, since food deprivation of our dogs may have reduced gastrointestinal absorption.

An aspect that cannot be determined with our data is the possibility that the pharmacokinetics of Sativex in dogs may present gender-dependent differences. Our study was carried out with a cohort of only 3 males and only 3 females, so too low numbers for identifying such differences. However, in our hands, the distribution of male and female data for the different parameters analyzed did not exhibit apparently any relevant differences. Anyway, the question would deserve further investigation with a greater cohort of males and females for both pharmacokinetic parameters and safety markers.

## 5. Conclusions

Our study demonstrated that single dose or a multiple dose sublingual administration of Sativex to naïve dogs was well-tolerated and produced the expected pharmacokinetic profile with maximal levels of phytocannabinoids detected at 1–2 h and suggested progressive accumulation after repeated treatment. In general, the pharmacokinetic properties demonstrated by Sativex in our study resemble many of the results published in previous pharmacokinetic studies with this phytocannabinoid-derived medicine in humans and laboratory rodents [34,35,36,37].

## References

[1] Chanda D., Neumann D., Glatz J.F.C. (2019). The endocannabinoid system: Overview of an emerging multi-faceted therapeutic target. Prostaglandins Leukot. Essent. Fatty Acids.

[2] Iannotti F.A., Di Marzo V., Petrosino S. (2016). Endocannabinoids and endocannabinoid-related mediators: Targets, metabolism and role in neurological disorders. Prog. Lipid. Res..

[3] Velasco G., Sánchez C., Guzmán M. (2015). Endocannabinoids and Cancer. Handb. Exp. Pharmacol..

[4] Howlett A.C., Johnson M.R., Melvin L.S. (1990). Classical and nonclassical cannabinoids: Mechanism of action--brain binding. NIDA Res. Monogr.

[5] Howlett A.C., Bidaut-Russell M., Devane W.A., Melvin L.S., Johnson M.R., Herkenham M. (1990). The cannabinoid receptor: Biochemical, anatomical and behavioral characterization. Trends Neurosci..

[6] Di Marzo V., Melck D., Bisogno T., De Petrocellis L. (1998). Endocannabinoids: Endogenous cannabinoid receptor ligands with neuromodulatory action. Trends Neurosci..

[7] Martin B.R., Mechoulam R., Razdan R.K. (1999). Discovery and characterization of endogenous cannabinoids. Life Sci..

[8] Pertwee R.G. (2008). Ligands that target cannabinoid receptors in the brain: From THC to anandamide and beyond. Addict. Biol..

[9] Pertwee R.G. (2012). Targeting the endocannabinoid system with cannabinoid receptor agonists: Pharmacological strategies and therapeutic possibilities. Philos. Trans. R. Soc. Lond. B Biol. Sci..

[10] Hill A.J., Williams C.M., Whalley B.J., Stephens G.J. (2012). Phytocannabinoids as novel therapeutic agents in CNS disorders. Pharmacol. Ther..

[11] Fernández-Ruiz J., Sagredo O., Pazos M.R., García C., Pertwee R., Mechoulam R., Martínez-Orgado J. (2013). Cannabidiol for neurodegenerative disorders: Important new clinical applications for this phytocannabinoid?. Br. J. Clin. Pharmacol..

[12] Keating G.M. (2017). Δ^9^-Tetrahydrocannabinol/Cannabidiol oromucosal spray (Sativex^®^): A review in multiple sclerosis-related spasticity. Drugs.

[13] Giacoppo S., Bramanti P., Mazzon E. (2017). Sativex in the management of multiple sclerosis-related spasticity: An overview of the last decade of clinical evaluation. Mult. Scler. Relat. Disord..

[14] Otero-Romero S., Sastre-Garriga J., Comi G., Hartung H.P., Soelberg Sørensen P., Thompson A.J., Vermersch P., Gold R., Montalban X. (2016). Pharmacological management of spasticity in multiple sclerosis: Systematic review and consensus paper. Mult. Scler..

[15] Maccarrone M., Maldonado R., Casas M., Henze T., Centonze D. (2017). Cannabinoids therapeutic use: What is our current understanding following the introduction of THC, THC:CBD oromucosal spray and others?. Expert Rev. Clin. Pharmacol..

[16] Sagredo O., Pazos M.R., Valdeolivas S., Fernandez-Ruiz J. (2012). Cannabinoids: Novel medicines for the treatment of Huntington’s disease. Recent Pat. CNS Drug Discov..

[17] Cristino L., Bisogno T., Di Marzo V. (2019). Cannabinoids and the expanded endocannabinoid system in neurological disorders. Nat. Rev. Neurol..

[18] Perez J., Ribera M.V. (2008). Managing neuropathic pain with Sativex: A review of its pros and cons. Expert Opin. Pharmacother..

[19] Valastro C., Campanile D., Marinaro M., Franchini D., Piscitelli F., Verde R., Di Marzo V., Di Bello A. (2017). Characterization of endocannabinoids and related acylethanolamides in the synovial fluid of dogs with osteoarthritis: A pilot study. BMC Vet. Res..

[20] Abramo F., Campora L., Albanese F., della Valle M.F., Cristino L., Petrosino S., Di Marzo V., Miragliotta V. (2014). Increased levels of palmitoylethanolamide and other bioactive lipid mediators and enhanced local mast cell proliferation in canine atopic dermatitis. BMC Vet. Res..

[21] Gesell F.K., Zoerner A.A., Brauer C., Engeli S., Tsikas D., Tipold A. (2013). Alterations of endocannabinoids in cerebrospinal fluid of dogs with epileptic seizure disorder. BMC Vet. Res..

[22] Fernández-Trapero M., Espejo-Porras F., Rodríguez-Cueto C., Coates J.R., Pérez-Díaz C., de Lago E., Fernández-Ruiz J. (2017). Upregulation of CB2 receptors in reactive astrocytes in canine degenerative myelopathy, a disease model of amyotrophic lateral sclerosis. Dis Model. Mech..

[23] Freundt-Revilla J., Heinrich F., Zoerner A., Gesell F., Beyerbach M., Shamir M., Oevermann A., Baumgärtner W., Tipold A. (2018). The endocannabinoid system in canine Steroid-Responsive Meningitis-Arteritis and Intraspinal Spirocercosis. PLoS ONE.

[24] Galiazzo G., Giancola F., Stanzani A., Fracassi F., Bernardini C., Forni M., Pietra M., Chiocchetti R. (2018). Localization of cannabinoid receptors CB1, CB2, GPR55, and PPARα in the canine gastrointestinal tract. Histochem Cell Biol..

[25] Averill D.R. (1973). Degenerative myelopathy in the aging German Shepherd dog: Clinical and pathologic findings. J. Am. Vet. Med. Assoc..

[26] Coates J.R., Wininger F.A. (2010). Canine degenerative myelopathy. Vet. Clin. N. Am. Small Anim. Pract..

[27] Awano T., Johnson G.S., Wade C.M., Katz M.L., Johnson G.C., Taylor J.F., Perloski M., Biagi T., Baranowska I., Long S. (2009). Genome-wide association analysis reveals a SOD1 mutation in canine degenerative myelopathy that resembles amyotrophic lateral sclerosis. Proc. Natl. Acad Sci. USA.

[28] Freundt-Revilla J., Kegler K., Baumgärtner W., Tipold A. (2017). Spatial distribution of cannabinoid receptor type 1 (CB1) in normal canine central and peripheral nervous system. PLoS ONE.

[29] Pirone A., Cantile C., Miragliotta V., Lenzi C., Giannessi E., Cozzi B. (2016). Immunohistochemical distribution of the cannabinoid receptor 1 and fatty acid amide hydrolase in the dog claustrum. J. Chem. Neuroanat..

[30] Chiocchetti R., Galiazzo G., Tagliavia C., Stanzani A., Giancola F., Menchetti M., Militerno G., Bernardini C., Forni M., Mandrioli L. (2019). Cellular distribution of canonical and putative cannabinoid receptors in canine cervical dorsal root ganglia. Front. Vet. Sci..

[31] Whalley B.J., Lin H., Bell L., Hill T., Patel A., Gray R.A., Elizabeth Roberts C., Devinsky O., Bazelot M., Williams C.M. (2019). Species-specific susceptibility to cannabis-induced convulsions. Br. J. Pharmacol.

[32] www.lgcgroup.com/pharma.

[33] Ash K., Hayes G.M., Goggs R., Sumner J.P. (2018). Performance evaluation and validation of the animal trauma triage score and modified Glasgow Coma Scale with suggested category adjustment in dogs: A VetCOT registry study. J. Vet. Emerg. Crit. Care.

[34] Karschner E.L., Darwin W.D., Goodwin R.S., Wright S., Huestis M.A. (2011). Plasma cannabinoid pharmacokinetics following controlled oral Δ^9^-tetrahydrocannabinol and oromucosal cannabis extract administration. Clin. Chem..

[35] Stott C.G., White L., Wright S., Wilbraham D., Guy G.W. (2013). A phase I study to assess the single and multiple dose pharmacokinetics of THC/CBD oromucosal spray. Eur. J. Clin. Pharmacol.

[36] Huestis M.A. (2007). Human cannabinoid pharmacokinetics. Chem. Biodivers..

[37] Johansson E., Norén K., Sjövall J., Halldin M.M. (1989). Determination of Δ^1^-tetrahydrocannabinol in human fat biopsies from marihuana users by gas chromatography-mass spectrometry. Biomed. Chromatogr..

